# Perforation in Typhoid Fever.— Successful Operation

**Published:** 1898-07-30

**Authors:** 


### PERFORATION IN TYPHOID FEVER.—SUCCESSFUL OPERATION.

Cases of successful operations for perforation of the intestine in typhoid fever are as yet but rare. Several points are worthy of note in a case recorded by Dr. Handford and Mr. Anderson, of Nottingham.[1] The operation was not done till 22½ hours after the occurrence of the perforation. The incision was made in the middle line; there was gas and fæculent matter in the peritoneum; the whole of the small intestine was deeply congested and covered with patches of lymph; the opening not being at first visible, the cæcum was found, and the ilium was traced up from it, the perforation being found within three feet; the orifice was about the size of a bean. The loop was pulled out of the abdomen, the opening was closed by Lembert's sutures of fine silk without any paring of the edges, the whole of the small intestines was then, loop by loop, pulled out, wiped with 1 in 2,000 biniodide solution, and replaced, the loins and pelvis then being treated in the same way. No douching was used. The abdominal wound was closed without drainage. During recovery there was an attack of suppurative nephritis, probably due to a septic infarction, otherwise the patient went on well. This case may be compared with one recorded in the last volume of the Medico-Chirurgical Transactions. This patient was under the care of Dr. Lauder Brunton, and was operated on by Mr. Bowlby. The opening in the gut was surrounded by a good deal of thickening, but it was successfully closed by Lembert's sutures. The abdomen was irrigated and the pelvis was drained. Recovery was uninterrupted. In a case reported at the same meeting which was under Dr. Herringham, and was also operated on by Mr. Bowlby, symptoms of perforation occurred during convalescence, and laparotomy was performed; no perforation, however, could be found, and the patient recovered. It was curious in this case how accurately the symptoms of perforation were simulated, and it is interesting to note that no harm followed the exploration. Which teaches a lesson. Of course, it would be easy enough for a careless operator to kill a patient by a mere exploration, and even a careful man may sometimes after opening the abdomen be tempted into some operative error, but we should be very much surprised if the records of our great hospitals for the last half-dozen years showed many cases in which a mere exploration performed properly and amid proper surroundings had done harm.

[1] Brit. Med. Jour., July 23.

